# Health Tract, No. 131

**Published:** 1865

**Authors:** 


Common lime quickly and perfectly absorbs carbonic and other disagreeable and unhealthful gases and odors; and for this purpose, in times of plagues, epidemics, and wasting diseases, is scattered plentifully in cellars, privies, stables, and gutters of the streets. It not only purifies the air and promotes physical health, but as a whitewash enlivens and beautifies wherever it is applied. As it is easily washed off by the rain if not properly prepared as a wash, it has to be so frequently reäpplied that it is considered troublesome by many; hence the rich use paint, and the poor use nothing to protect their dwellings, fences, etc., from the ravages of the weather; yet the difference between a well-whitewashed farm and one where no lime is used, would amount to a large per centage in case of a sale. For the physical and moral benefits which may arise from the abundant use of lime as a whitewash, several modes of preparing it, so as to make it more durable, whether applied in-doors or out, are here given, with the suggestion that the same amount of money necessary to keep a man's premises well whitewashed, can not be expended to as great a moral and healthful advantage in any other way.

1. One ounce of white vitriol (sulphate of zinc) and three ounces of common salt to every four pounds of good fresh lime, that is, lime which has not fallen into dry powder from exposure to the atmosphere, with water enough to make it sufficiently thin to be applied with a brush, makes a durable out-door whitewash.

2. Take a clean water-tight barrel, or other wooden cask, and put into it half a bushel of lime in its rock state, pour enough boiling water on it to cover it five inches deep, and stir it briskly until it is dissolved or thoroughly "slacked," then put in more water and add two pounds of sulphate of zinc—that is, white vitriol—and one pound of common salt; these harden the wash and prevent cracking; this may be colored according to taste by adding three pounds of yellow ochre for a cream color; four pounds of umber for a fawn color, with a pound each of Indian red and lamp-black.

3. Mix up half a pail of lime and water ready for whitewashing; make a starch of half a pint of flour and pour it, while hot, into the lime-water while it is hot. This does not rub off easily.

4. A good in-door whitewash for a house of six or eight rooms is made thus: take three pounds of Paris white and one pound of white glue; dissolve the glue in hot water, ànd made a thick wash with the Paris white and hot water, then add the dissolved glue and sufficient water to make it of the proper consistence for applying with a brush. If any is left over, it hardens by the morning; but it may be dissolved with hot water; still it is best to make only enough to be used each day; spread it on while it is warm.

It is said to add to the value and lastingness of any lime-wash if the vessel in which it is slacking is kept covered with a cloth; this not only confines the heat, but keeps the very finest of the particles of lime from being carried off by steam, wind, or otherwise.

When it is taken into account how much buildings and fences are protected against the destructive influences of the weather, if they are plentifully whitewashed in April and November, to say nothing of the cheeriness, beauty, and purity which it adds to any dwelling, it is greatly to be desired that the practice of whitewashing liberally twice a year should be adopted by every household in the nation, where paint can not be afforded, and on every farm.

